# Management of a bilateral mandibular fracture in a single-humped camel 

**Published:** 2017-06-15

**Authors:** Hadi Imani Rastabi, Abdolvahed Moarabi, Ahmad Khajeh, Narges Kavosi

**Affiliations:** *Department of Clinical Science, Faculty of Veterinary Medicine, Shahid Chamran University of Ahvaz, Ahvaz, Iran*

**Keywords:** Anesthesia, Camel, Mandibular fracture, Wire fixation

## Abstract

In the present case report, the comprehensive management of a bilateral mandibular fracture in a single-humped camel including pre-, peri- and post-operative cares is described. A one-year-old camel with the overhanging of the rostral part of the lower jaw which occurred seven days ago was presented. After clinical and radiographic examinations, a bilateral mandibular fracture at the caudal part of canine teeth was diagnosed. It was decided to repair the fracture surgically under general anesthesia. The camel was restrained in sitting position and was remained in this position during anesthesia and surgery period. After premedication with acepromazine (0.10 mg kg^-1^) and xylazine (0.20 mg kg^-1^) intramuscularly, anesthesia was induced using ketamine and diazepam intravenously (2.00 and 0.10 mg kg^-1^, respectively). Maintenance of anesthesia was performed by repeated doses of xylazine and ketamine intravenously (0.10 and 1.00 mg kg^-1^, respectively) as needed. After preparation of the oral cavity, the fracture was reduced and an interfragmentary wire and an interdental wire (1.00 mm diameter size) were applied on each side of the mandible for the fixation of fracture segments. After two months, the interdental wires were removed while the interfragmentary wires were left intact. Follow up the camel three months after surgery, showed the full ability of prehension and chewing of roughages by the camel.

## Introduction

Mandibular fractures are the most common types of fractures in camels which often occur in the rut males following fighting with each other. These fractures are mainly seen across first premolar teeth or at the cranial or caudal part of interdental space.^1^^,^^2^ The presence of mental canal and alveoli of the first premolar teeth and relatively small cross-sectional diameter make this region susceptible to fracture.^1^^-^^3^ Because of high value and effective role of camel in some areas, optimal management and reducing the complications related to these fractures can be important. In the present case report, the management of a bilateral mandibular fracture including history, signs, diagnosis, perioperative care, anesthesia and fixation by concurrent use of interfragmentary and interdental wiring in a single-humped camel is described.

## Case Description

**Fig. 1:**
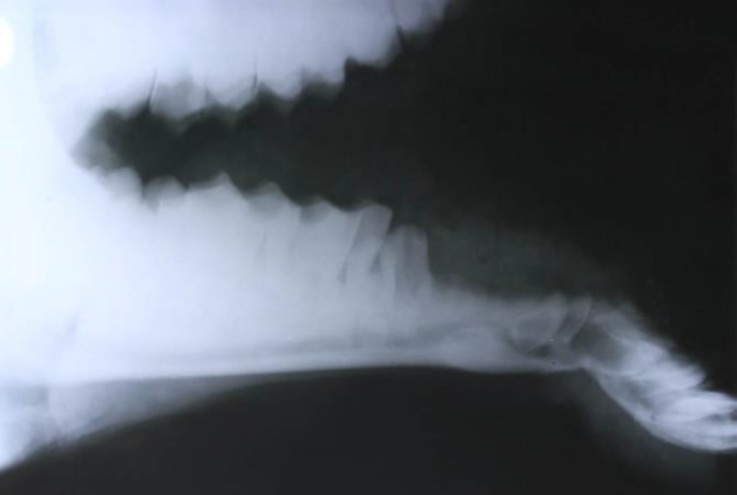
Transverse mandibular fracture at the caudal part of canine teeth

A one-year-old male camel with the overhanging of the lower jaw was referred to the Hospital of Faculty of Veterinary Medicine, Shahid Chamran University of Ahvaz, Ahvaz, Iran. Based on the owner’s statement, the camel had been suffered during fighting with another camel seven days ago and the frontal aspect of the lower jaw was deviated ventrally. The animal was not able to grasped foods and had been nourished manually. In the physical examination, bilateral separation of the mandible and lacerations at alveolar and oral gingiva were seen. Radiography at lateral views revealed a transverse bilateral mandibular fracture at the caudal part of canine teeth (Fig. 1). It was decided to repair the fractures surgically under general anesthesia.

**Fig. 2:**
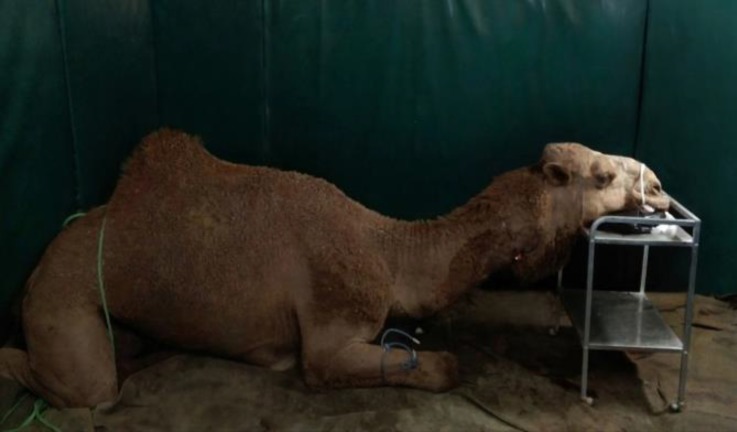
Restraining and maintaining the camel in sitting position for surgery.

After about 18 hr fasting, the animal was transferred to the surgery theater, and restrained in sitting position with tightening ropes on the feet (Fig. 2). After acclimatizing to the circumstances, the camel was premedicated with ace-promazine (0.10 mg kg^-1^, Alfasan, Woerden, The Netherlands) intramuscularly (IM) into the mass of quadriceps muscle. Twenty min later xylazine (0.20 mg kg^-1^, Alfasan) was administered IM.

When the animal was sufficiently sedated, right jugular vein was catheterized using a 14-gauge intravenous (IV) catheter. Anesthesia was induced, 20 min after xylazine administration, with combination of ketamine (2 mg kg^-1^, Alfasan) and diazepam (0.10 mg kg^-1^, Caspian Tamin, Rasht, Iran) intravenously. Lidocaine 1.00% with epinephrine (20 mL, Aburaihan, Tehran, Iran) was also infiltrated at the oral and alveolar sides of gingival mucosa. Anesthesia was maintained during the procedure by repeated doses of xylazine and ketamine IV at half doses for induction (0.10 and 1.00 mg kg^-1^, respectively) as needed. During the anesthesia, the animal was maintained in sitting position, the neck of the camel was stretched and the head was placed on a surgical trolley. Heart and respiratory rates and palpebral and corneal reflexes were checked regularly.

**Fig. 3:**
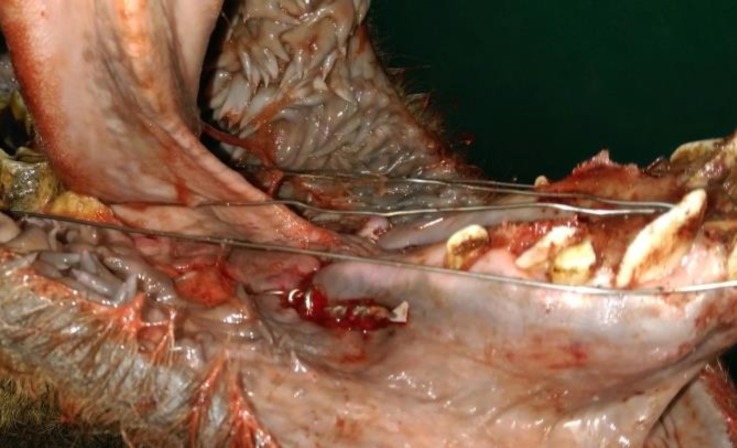
Interfragmentary and interdental wiring of the mandibular fracture of the camel.

**Fig. 4:**
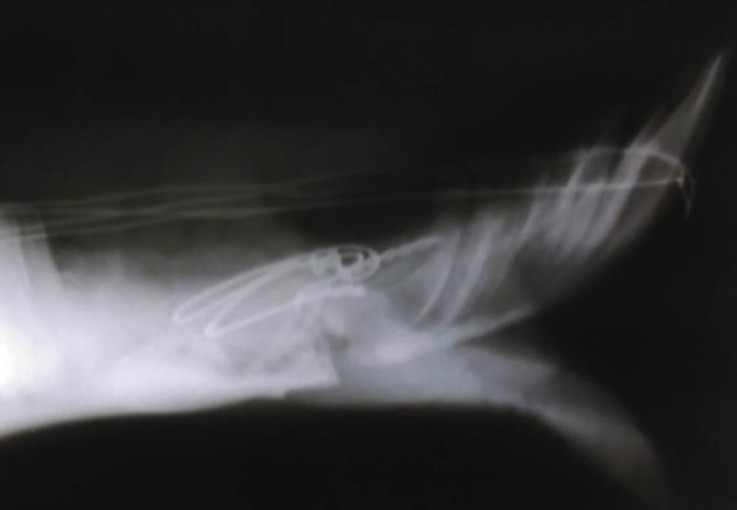
Postoperative radiography of repaired mandibular fracture using orthopedic wire.

The oral cavity of the animal was washed with normal saline and diluted povidone iodine solution copiously and the foods, clots and debris were removed. The fracture was reduced and the mandible was maintained in its proper position. Using an electrical drill, two holes were created on either side of the fracture line. Care was taken to avoid dental roots and to maintain proper distance from fracture line. An orthopedic wire (1.00 mm diameter; Aesculap, Melsungen, Germany) was passed through the both holes and was tightened at the lateral side of the mandible. After applying the wires on both sides, the orthopedic wire was passed between the first and second right cheek teeth and central incisors and twisted around the teeth toward the root of incisors at the cranio-lateral side of the mandible. The same procedure was performed at the opposite side (Fig. 3). No instability or malocclusion was noted after fixation. After accomplishing the procedure, the oral cavity was washed with normal saline. For more support, a circular bandage was applied around the muzzle. Postoperative radiographic evaluation revealed satisfactory anatomical alignment of the fracture (Fig. 4). The animal was recovered uneventfully and no complications such as regurgitation or delirium were observed during anesthesia and recovery period.

For postoperative care penicillin-streptomycin (30,000 IU kg^-1^ penicillin and 10 mg kg^-1^ streptomycin; Bayer Aflak, Azna, Iran) was administered IM daily for five days. Intramuscular ketoprofen (2 mg kg^-1^; Razak, Tehran, Iran) was administered for three days. The animal was allowed free access to water but feeding was recommended to be done manually with semisolid foods for at least two weeks. Feeding and drinking, during bandage application, were done by creating a small space between jaws without removing the supporting bandage. Daily washing of oral cavity and confining the animal in a closed area were also advised. After two months, the animal was sedated with xylazine (0.20 mg kg^-1^, IM) and interdental wires were removed while interfragmentary wires were left intact. On manual palpation, the fractured segments of the mandible had been tightly fixed, however, a slight ventral deviation of the lower jaw was noticeable. Follow-up the animal three months after surgery showed the full ability of the camel to prehend and to chew roughages. No complication was reported by the owner.

## Discussion

This case report described the management of a bilateral mandibular fracture in a single-humped camel with emphasis on anesthesia, fixation method and postoperative care. Although mandibular fractures have been reported in camels, the authors did not find a comprehensive study describing various aspects of an efficient management of these fractures in camels during pre-, peri-, and post-operative care periods.

Administration of premedication and anesthetic agents to a camel may be encountered by difficulties especially when the animal is not cooperative. It is best that the animal to be restrained by the owner’s contribution with roping the legs and in sitting position. Covering the face of the animal to avoid biting or spitting was also recommended.^4^ The doses used for premedication, induction and maintenance of anesthesia in the current case were documented elsewhere.^4^^,^^5^ Because of lack of proper-sized tracheal tube and remained swallowing reflex, intubation was not attempted in the current case, however, to prevent possible problems, the animal was maintained in sternal recumbency and the head of the camel was held up by putting a surgical trolley under the head, during anesthesia and surgery period. Monitoring of the anesthetized animal was performed by recording heart rate, respiratory rate and evaluation of palpebral and ocular reflexes during the anesthesia that were in the normal limits based on previous reports.^4^

Different methods have been employed for fixation of mandibular fractures in camels including interdental wiring,^3^^,^^6^ U-bar application,^7^ combination of cross pin fixation and tension band wires,^8^ bone plating^9^ and different types of plaster of Paris bandage.^3^^,^^10^ Based on the location, time elapsed to the referral and severity of the fracture, a method or a combination of methods could be used. In the current case, because of some concerns in transporting the animal and providing more stable fixation, the combination of interfragmentary and interdental wiring was used. Applying a circular bandage was performed to support the healing at first days after surgery. Healing of mandibular fracture is relatively rapid and the time for camel’s mandibular fracture has been reported in the range of 7 to 12 weeks.^1^^,^^7^ In the present case, Interdental wires were removed after two months post operation, but interfragmentary wires were left intact. The latter wires were relatively embedded under the oral mucosa and did not annoy the animal. At this time, the fracture site was stable and no movement was seen on manual palpation. Since buried cerclage wires are not removed unless infected,^11^ the interfragmentary wires were not removed.

Wire loosening, development of submandibular abscesses, ventral malalignment, buccal infection, intraoral ulceration, and osteomyelitis have been reported as potential complications after surgical repair of mandibular fractures in camels.^2^^,^^7^^,^^12^ In the present case no notable complications were observed except for a slight ventral deviation of the fractured fragment. It seems that the most adverse effects associated with these fractures can be attributed to the trauma and contamination of the surgical area. Thus, restricting the animal, regular oral washing and perioperative antibiotic therapy, which were performed in the current case, can prevent these postoperative complications. The ventral deviation, might have been caused due to poor post-operative cares by the owner, however, ventral deviation is reported as a common outcome in mandibular fracture fixed by interdental wiring.^2^
